# Emergency operation for spontaneous spinal epidural hematoma in a patient with severe back pain, which made it difficult to evaluate neurological deficits: a case report

**DOI:** 10.1186/s40981-019-0246-x

**Published:** 2019-03-20

**Authors:** Hironao Matsuda, Chiaki Nemoto, Takumi Sekine, Katsuhiko Sato, Youichi Tanaka, Masahiro Murakawa

**Affiliations:** 1 The Junior Resident Center, Ohara General Hospital, Fukushima, Japan; 2 Department of Anesthesiology, Ohara General Hospital, Fukushima, Japan; 3 Department of Orthopaedics, Ohara General Hospital, Fukushima, Japan; 4 Department of Anesthesiology, Fukushima Medical University School of Medicine, Fukushima, Japan

**Keywords:** Spontaneous spinal epidural hematoma, Acute back pain, Neurological deficit

To the editor

Spontaneous spinal epidural hematomas (SSEH) are relatively rare and are generally characterized by sudden back pain followed by neurological deficits [1–3]. In some patients without neurological deficits, diagnosing SSEH is difficult. We discuss a patient with SSEH with severe back pain, which made it difficult to evaluate neurological deficits, in the emergency room (ER).

## Case presentation

**Fig. 1:**
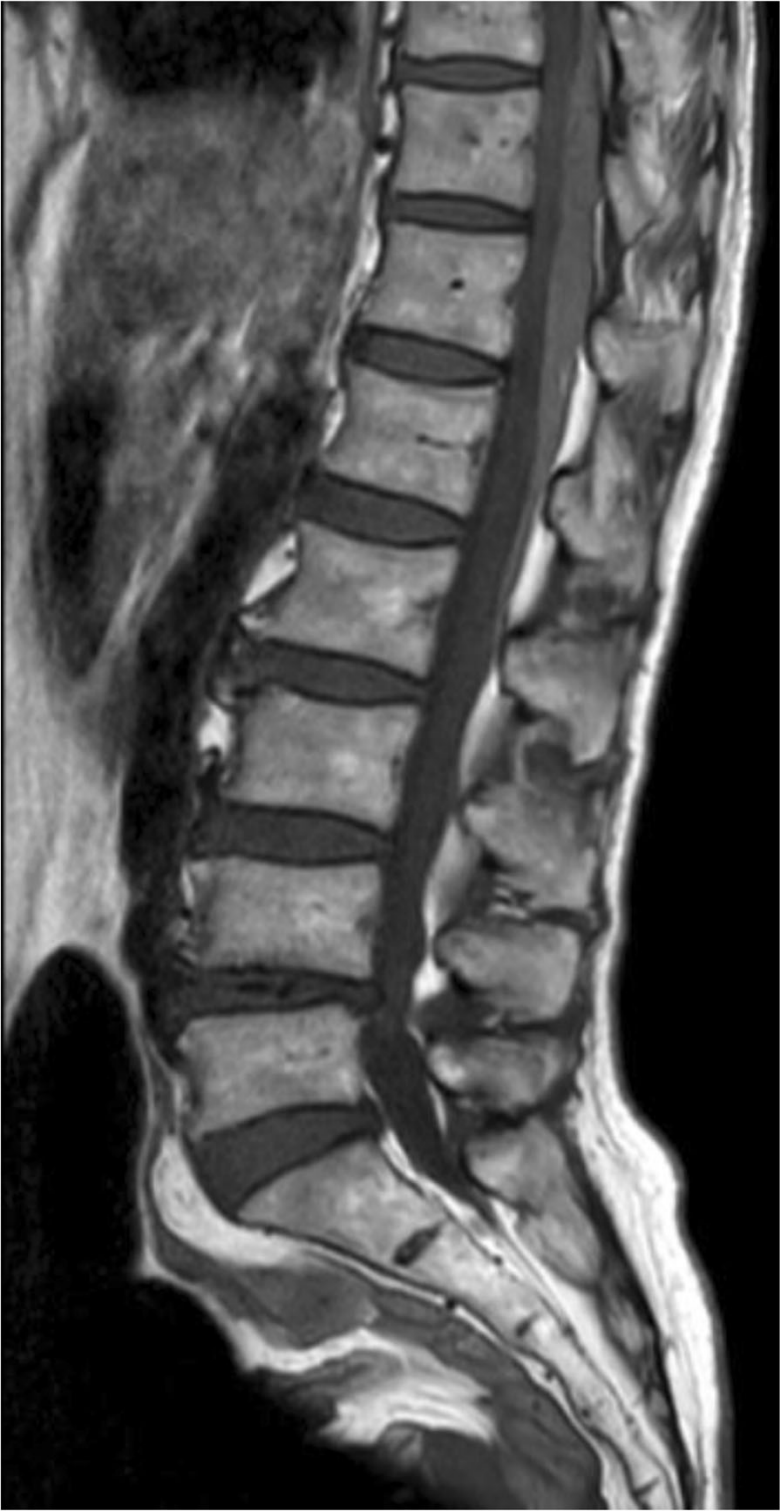
T1-weighted image of the patient’s spine. T1-weighted magnetic resonance image showing compression of the posterior aspect of the spinal cord by a hematoma extending from T10–L1

A 70-year-old woman, 157-cm tall and weighing 40 kg, experienced acute back pain upon waking and called emergency medical services. Her medical history included only hypertension, for which she was taking 10-mg manidipine hydrochloride each morning and no anticoagulants. On presentation, her consciousness level was clear, blood pressure 176/93 mmHg, heart rate 120 beats/min, SpO_2_ 100%, and respiratory rate 30 breaths/min. Abdominal ultrasonography did not indicate abnormal findings, and blood biochemical parameters, including coagulation tests, were normal. Although manual motor testing was difficult to perform because of the patient’s severe back pain, no obvious neurological deficits were confirmed. The patient’s pain numerical rating score was 9–10; therefore, we administered 600 mg of acetaminophen and inserted a 25-mg diclofenac suppository. Twenty hours after onset, her back pain had almost disappeared, but she had developed weakness in her lower extremities (Table 1) and sensory disorder in the lower umbilical region. We performed magnetic resonance imaging (MRI) immediately, which showed compression of the posterior aspect of the spinal cord by a hematoma extending from T10–L1 (Fig. 1). Emergency evacuation of the hematoma, T10–T12 total laminectomy, and L1 laminotomy were performed immediately. Her postoperative course was good, and she was discharged from the hospital 17 days postoperatively without complications.

**Table 1:** Results of manual muscle testing

Muscle	Right	Left
Iliopsoas muscle	4−	4
Quadriceps muscle	4+	5−
Tibialis anterior muscle	4	5−
Extensor hallucis longus muscle	4	4
Extensor digitorum muscle	4	4
Gastrocnemius muscle	5	5
Flexor hallucis longus muscle	5−	5
Flexor digitorum longus muscle	5	5

*5* holds test position against maximal resistance, *5−* holds test position against slight to maximal resistance, *4+* holds test position against moderate to strong pressure, 4 holds test position against moderate resistance, *4−* holds test position against slight to moderate pressure

## Discussion

SSEHs are difficult to diagnose in patients without apparent neurological deficits [4], and severe back pain makes it difficult to detect subtle neurological findings. The incidence of SSEH is 0.1 per 100,000 individuals [5] and is 1.4 times higher in men than in women [3]. One of the possible risks of SSEH is uncontrolled hypertension [6]. Hypertension was present in our patient, and although her blood pressure was high at admission because of the severe back pain, her blood pressure was usually well controlled. Generally, most cases of SSEH are idiopathic [7].

The most common treatment for SSEH in patients with neurological deficits is surgical evacuation of the hematoma [8, 9]. The mortality rate associated with this operation is low [10]; therefore, surgical evacuation of the hematoma should be the first choice for SSEH with neurological deficits. In our patient, severe back pain masked neurological deficits, making it difficult to diagnose SSEH. Additionally, pain-related symptoms, including a high respiratory rate, make assessing neurological findings confusing. We considered operation instead of conservative treatment because of the progression of our patient’s neurological deficits and the size of the hematoma seen on MRI.

Acute back pain is a common symptom in the ER, and mild neurological deficits may not be recognized at presentation with concurrent severe back pain. MRI is the most useful method to diagnose SSEH, and surgical intervention provides a good neurological prognosis.

## Abbreviations

ER: Emergency room
MRI: Magnetic resonance imaging
SSEH: Spontaneous spinal epidural hematoma
